# Genetic polymorphism of ACE and the angiotensin II type1 receptor genes in children with chronic kidney disease

**DOI:** 10.1186/1476-9255-8-20

**Published:** 2011-08-23

**Authors:** Manal F Elshamaa, Samar M Sabry, Hafez M Bazaraa, Hala M Koura, Eman A Elghoroury, Nagwa A Kantoush, Eman H Thabet, Dalia A Abd-El Haleem

**Affiliations:** 1 Pediatric Department, National Research Centre, Cairo, Egypt; 2 Pediatric Department, Faculty of Medicine, Cairo University, Cairo, Egypt; 3 Clinical & Chemical Pathology Department, National Research Centre, Cairo, Egypt

**Keywords:** angiotensin-converting enzyme, angiotensin II type one receptor, DNA polymorphisms, end-stage renal disease, Children

## Abstract

**Aim and Methods:**

We investigated the association between polymorphisms of the angiotensin converting enzyme-1 (ACE-1) and angiotensin II type one receptor (AT1RA1166C) genes and the causation of renal disease in 76 advanced chronic kidney disease (CKD) pediatric patients undergoing maintenance hemodialysis (MHD) or conservative treatment (CT). Serum ACE activity and creatine kinase-MB fraction (CK-MB) were measured in all groups. Left ventricular mass index (LVMI) was calculated according to echocardiographic measurements. Seventy healthy controls were also genotyped.

**Results:**

The differences of D allele and DI genotype of ACE were found significant between MHD group and the controls (p = 0.0001). ACE-activity and LVMI were higher in MHD, while CK-MB was higher in CT patients than in all other groups. The combined genotype DD v/s ID+II comparison validated that DD genotype was a high risk genotype for hypertension .~89% of the DD CKD patients were found hypertensive in comparison to ~ 61% of patients of non DD genotype(p = 0.02). The MHD group showed an increased frequency of the C allele and CC genotype of the AT1RA1166C polymorphism (P = 0.0001). On multiple linear regression analysis, C-allele was independently associated with hypertension (P = 0.04).

**Conclusion:**

ACE DD and AT1R A/C genotypes implicated possible roles in the hypertensive state and in renal damage among children with ESRD. This result might be useful in planning therapeutic strategies for individual patients.

## Background

Chronic kidney disease (CKD) is a complex disorder encompassing a large variety of phenotypes. Each phenotype is a result of an underline kidney disease and superimposing environmental and genetic factors. The complexity of the phenotypic makeup of renal diseases makes it difficult to diagnose and predict their progression and to decide on the optimal treatment for each patient. End stage renal disease (ESRD) is an advanced form of chronic renal failure where renal function has declined to approximately 10% of normal prior to initiation of dialysis or transplantation [1]. The impact of genetic variability on the development of renal failure is becoming clearer and emphasizes the need to elucidate the genetic basis for renal diseases and its complications. Renal functions and blood pressure are tightly linked. Physiologically, kidneys provide a key mechanism of chronic blood pressure control [1], whereas elevated blood pressure affects renal function via pressure naturesis mechanism [2,3]. Patho-physiologically, long standing hypertension attenuates pressure naturesis [4] and can cause or at least contribute to renal damage [5]. Therefore, hypertension is one of the imperative contributing factors associated with both causation and progression of renal failure [6-8].

The Renin-angiotensin system (RAS) is a key regulator of both blood pressure and kidney functions and may play a role in their interaction. Its role in the pathogenesis of hypertension is well documented, but its contribution to chronic renal failure, progression of kidney nephropathy is still debated [9]. It has been seen that RAS blockers i.e. both angiotensin converting enzyme (ACE) inhibitors and angiotensin receptor blockers lower blood pressure and can also attenuate or prevent renal damage [10]. However, major inter-individual treatment responses to RAS inhibitors have been noted [11] and it remains difficult to predict responders based on known patho-physiological characteristics [12]. In such a situation, genetic variability in the genes of different components of RAS is likely to contribute for its heterogeneous association in the renal disease patients. Angiotensin converting enzyme-1 (ACE-1) is an important component of RAS and it determines the vaso-active peptide angiotensin-II. Its inhibition reduces the pace of progression of the majority of chronic nephropathies [13,14]. Among the candidate genes of the RAS, the ACE, and angiotensin II type 1 receptor (AT1RA1166C) genes seem to be particularly biologically and clinically relevant to renal disease. The genetic polymorphisms of these key components of RAS provide a basis for studying the relationship between genetic variants and the development of vascular and/or renal damage in individual subjects [15,16].

The gene coding for ACE is subjected to an insertion/deletion (I/D) polymorphism that is a main determinant of plasma and tissue ACE levels [17]. The D allele has been linked to a failure of the reno-protective action of ACE inhibitors to retard the development of ESRD [18,19].

Several polymorphisms were identified in the AT1RA1166C gene which was linked to essential hypertension [20]. It has been considered a risk factor for hypertension and cardiovascular (CVD) disease [21].

The aim of the present study was to investigate the association between polymorphisms of the ACE and AT1RA1166C genes and the occurrence of renal disease in 76 advanced CKD (stages 4 and 5) pediatric patients undergoing MHD or CT. In addition, we evaluated the prevalence and the severity of left ventricular hypertrophy (LVH) and its association with these genetic polymorphisms.

## Methods

### Study populations

Seventy six Egyptian pediatric patients with advanced CKD [stages 4 and 5 based on estimated glomerular filtration rate (e-GFR) according to the National Kidney Foundation classification [22] were included in the study. They were divided into two groups undergoing CT (n = 32) or MHD (n = 44). MHD children were selected from the hemodialysis unit of the Center of Pediatric Nephrology and Transplantation (CPNT), while CT children were selected from the Nephrology pediatric clinic, Children's Hospital, Cairo University. The study was done from March 2009 to December 2009. In CT patients the causes of renal failure were renal hypoplasia or dysplasia (n = 14), obstructive uropathies (n = 8), neurogenic bladder (n = 4), not known (n = 4), and metabolic (n = 2). In MHD, the causes of renal failure were: hereditary nephropathies (n = 17), obstructive uropathies (n = 6), neurogenic bladder (n = 2), glomerulopathy (n = 2), renal hypoplasia or dysplasia (n = 2), and unknown causes (n = 15). The inclusion criteria for MHD patients included a constantly elevated serum creatinine level above the normal range (ranging from 3.4 to 15.8 mg/dl) and were dialysed for not less than 6 months. They were treated with hemodialysis for 3-4 h three times weekly with a polysulfone membrane using bicarbonate-buffered dialysate. The Duration of hemodialysis was 2.82 ± 1.37 years. Thirty one MHD patients and 16 CT patients were taking anti-hypertensive treatment. The following classes of drugs were employed: *α*-adrenoceptor antagonists in one MHD and two CT, *ß*-blockers in nine MHD, ACE inhibitors in seventeen MHD and six CT, and Ca channel blockers in twenty-nine MHD and ten CT. Subjects were taking their medication when ACE activity was measured and no influence of medication on the measurement. In 1967, Ng and Vane [2] showed that the plasma (ACE) is too slow to account for the conversion of angiotensin I to angiotensin II *in vivo*. Subsequent investigation showed that rapid conversion occurs during its passage through the pulmonary circulation [10].

To control for differences in age and body size, blood pressure were indexed to the age, gender and height-specific 95^th ^percentile for each subject (measured systolic (SBP) or diastolic blood pressure (DBP) was divided by the age-gender- and height- specific 95^th ^percentile). Hypertension was defined as indexed SBP or DBP ≥ 1.0. None of CKD patients had cardiovascular events on the basis of examination and detailed clinical history.

All control subjects (n = 70) were healthy with no clinical signs of vascular or renal disease and no family history of renal disease as assessed by medical history and clinical examination, as well as a lack of medications taken at the time of the study. Healthy control subjects were selected to be matched for age and gender to the patient groups, as well as within the same BMI limits. They were collected from the pediatric clinic (A part from the Medical Services Unit) of National Research Centre (NRC) which is one of the biggest research centres in Egypt. An informed consent for genetic studies was obtained from parents of all participants. The protocol of the study was read and approved by the Ethics Committee of NRC in Egypt.

### -Biochemical markers

Venous blood samples were collected in the morning after an overnight fast on a midweek dialysis day, before the dialysis session. Three ml of venous blood sample was collected in EDTA vials for the extraction of genomic DNA. Pre- and post-dialysis kidney function test were determined by standard laboratory methods. Estimations of the plasma concentration of total cholesterol (TC), triglyceride (TG) and HDL cholesterol were made by using an Olympus AU400 (Olympus America, Inc., Center Valley, Pa., USA).

For determination of cardiac markers, MB fraction of creatine kinase (CK-MB) was measured by ELISA assay (Monobind Inc., Lake Forst, CA92630, Product code: 2925-300, USA) [23].

The determination of high sensitivity C-reactive protein (hs-CRP) in serum was performed by solid-phase chemiluminescent immunometric assay (Immulite/Immulite 1000; Siemens Medical Solution Diagnostics, Eschborn, Germany) [24].

The detection of ACE activity in serum was done by a kinetic colorimetric determination via FAGG (N-[3-(2-furyl) acryloyl]-L-phenylalanylglycylglycine) method. (Biochemical enterprise). The ACE presented in the serum catalyzes the hydrolysis of the FAGG; forming furyl acryloyl phenylalanine (FAP). The decrease of the absorbance in the unit time at 340 nm is proportional to the activity of the ACE in the serum [25].

### -Determination of genotypes

DNA was extracted from whole blood using a QIAamp Blood mini-prep Kit (QIAGEN, Germany). ACE I/D genotype was determined according to the method of losiro et al. [26]. Each DD genotype was confirmed by using insertion-specific primers. The products were of the size 190 bp and 490 bp for I and D allele respectively. Hence, single bands of 190 and 490 bp confirmed homozygous II and DD genotypic state respectively, whereas two bands of 190 and 490 bp confirmed heterozygous ID genotype. To examine the human AT1RA1166C variant sequences 25 pmol of primers were used in a total 25 μl volume. There was an initial denaturation at 94°C for 10 min. followed by 35 cycles of 1 min at 94°C, 1 min. at 55°C and 1 min at 72°C, final extension was at 72°C for 10 min. The PCR products were digested with 5 μ of restriction enzyme DdeI and visualized on 2% agarose gels stained with ethidium Bromide [26].

-Echocardiographic imaging was performed using the Vivid 3 Pro machine (Norway) equipped with 3 and 7 MHz transducers. Two dimensional (2D) guided M-mode measurements were made in supine position. Left ventricular mass (LVM) was calculated using measurements made according to the recommendations of the American Society of Echocardiography: LVM = 0.8 [1.04 ([LVEDD+PWT+IVST]^3^-[LVEDD]^3^)]+ 0.6 g, where LVEDD is left ventricular diameter in end diastole, PWT is posterior wall thickness in diastole, and IVST is inter-ventricular septum thickness in end diastole. The calculated mass correlated well with necropsy values for LVM [27]. Left ventricular mass index (LVMI) was calculated as LVM divided by height (meters) ^2.7^. Correcting LVM for height^2.7 ^minimizes the effect of gender, age, and obesity [28]. Severe LV hypertrophy was defined as LVMI greater than 51 g/m^2.7^, which has been shown to be at four- fold greater risk of cardiovascular morbid outcome in adult patients with hypertension [29]. This value is above the 99^th ^percentile for LVMI in normal children and adolescents [28]. Echocardiographic measurements were performed on non-dialysis days for MHD patients and on routine clinic visits for CT patients.

### Statistical analysis

Statistical package for social science (SPSS) program version 11.0 was used for analysis of data. Data were summarized as mean ± SD, range or percentage. Histograms and normality plots were used for evaluating the normality of data. For those data with skewed distribution, log transformation was performed before a t-test. Power analysis was used to calculate the minimum sample size required to accept the outcome of a statistical test with a particular level of confidence. A sample size of 20 will give us approximately 80% power (alpha = 0.05, two-tail) to reject the null hypothesis of zero correlation. We used power calculations performed by the Power and Precision program (Biostat) to determine the number of chromosomes required to detect a significant difference between the polymorphism frequency in the reference population and the expected frequency. Power commonly sets at 80%; however, at that level, a polymorphism would be missed 20% of the time. Data were valuated between the experimental groups by One-Way Analysis of Variance (ANOVA) followed by Tukey's multiple comparison test. Allele and genotypic frequencies for ACE and AT1R alleles were calculated with the gene counting method. Hardy-Weinberg equilibrium was tested by using the Pearson Chi-square *(X^2^*) test. A 2 × 2 contingency table was used for test of the differences of allele frequencies between cases and controls. Odds ratios (OR) with 95% confidence intervals (CI) were estimated for the effects of high risk alleles. Clinical characteristics of CKD patients with different ACE and AT1R genotypes were compared using independent t test. Pearson's analysis was performed to correlate LVMI with the individual variables. Multiple regression analysis was performed to assess the combined influence of variables on hypertension and LVMI values. A p value of < 0.05 was considered statistically significant.

## Results

Anthropometric, clinical and biochemical parameters in controls and CKD subjects are shown in (Table 1)

**Table 1:** Various parameters in children with chronic kidney disease and control subjects

	CT (n = 32)	MHD (n = 44)	Controls (n = 70)	P value
**Age(Years)**	9.14 ± 7.59	10.62 ± 3.49	10.7 ± 4.51	0.14

**Gender (M/F)**	15 (46.88%)/17(53.12%)	24(54.55%)/20(45.45%)	40(57.14%)/30(42.86%)	0.30

**BMI (kg/m^2^)**	17.64 ± 1.17	18.89 ± 3.00	20.60 ± 1.44	0.71

**SBP (mmHg)**	98.66 ± 6.66	125.13 ± 16.36 ^b^*	95.54 ± 9.70	0.01

**Indexed SBP**	0.90 ± 0.85	1.04 ± 0.14 ^b^**	0.73 ± 0.05	0.001

**DBP (mmHg)**	64.66 ± 6.67	83.13 ± 12.76 ^b^*	61.55 ± 10.10	0.01

**Indexed DBP**	0.90 ± 0.0.86	1.00 ± 0.10 ^b^**	0.72 ± 0.05	0.001

**Creatinine** **(mg/dl)**	3.93 ± 3.75 ^a^*	6.30 ± 1.45 ^b^**	0.73 ± 0.33	0.002

**Predialysis urea, (mg/dl)**	51.12 ± 10.45 ^a^*	70.56 ± 19.61 ^b^*	7.76 ± 2.53	0.02

**e-GFR, ml/min/1/1.73 m2**	15.41 ± 1.76 ^a^**	11.30 ± 3.35 ^b^**	86 ± 8.8	0.003

**Dialysis, Yrs**		2.73 ± 1.58		

**Kt/V**		1.68 ± 0.40		

**Total cholesterol** **(mg/dl)**	164.44 ± 50.10^ac^**	192.04 ± 50.37 ^b^*	161.31 ± 18.75	0.06

**Triglycerides** **(mg/dl)**	160.78 ± 57.33 ^a^**	146.00 ± 65.98 ^b^**	63.31 ± 17.35	0.001

**HDL- cholesterol (mg/dl)**	21.35 ± 1.17^a^*	27.33 ± 9.87 ^b^*	40.55 ± 7.83	0.01

**hs-CRP** **(mg/dl)**	3.04 ± 3.24	3.62 ± 3.97 ^b^*	1.35 ± 0.65	0.04

**CK-MB (ng/ml)**	6.23 ± 2.46 ^a^*	5.26 ± 1.14	4.20 ± 0.20	0.04

**ACE-activity(IU/l)**	53.02 ± 22.44	70.47 ± 53.73 ^b^**	30.11 ± 8.85	0.03

**Left ventricular mass index (g/m^2.7^)**	49 ± 5.20 ^a^*	52.86 ± 10.10 ^b^*	35.10 ± 8.12	0.04

**Severe left ventricular hypertrophy, n (%)**	6(18.75%)	25(56.82%)		

Data was evaluated by ANOVA test. Values were presented as means ± SD or percentage as applicable. CT = conservative treatment, MHD = maintenance hemodialysis, ACE = angiotensin converting enzyme, BMI = body mass index, SBP = systolic blood pressure, DBP = diastolic blood pressure, eGFR = estimated glomerular filtration rate, Kt/V = adequacy of hemodialysis, hs-CRP = high sensitivity C-reactive protein, CK-MB = creatine kinase-MB fraction. ^a^*P < 0.05 or ^a^**P < 0.01 vs. controls and CT^b^, *P < 0.05 or ^b^**P < 0.01 vs. controls and MHD and, ^c^P < 0.05 vs. CT and MHD.

### Distributions of ACE and AT1R genotypes

Independent segregation of alleles for these studied polymorphisms was kept in HWE. Genetic association analyses with Pearson Chi-square test was performed and data are summarized in Table 2.

**Table 2:** Distribution of alleles and gene polymorphisms in CKD patients and in controls

Gene		CT (n = 32)	MHD (n = 44)	Controls (n = 70)	Significance
**ACE Alleles**	I	24 (37.5%)	20(22.73%)	97 (69.29%)	*For D allele MHD Carriers: OR = 0.13, 95% CI (0.07-0.24) *X^2 ^= 46.89*, *P = 0.0001*
		
	D	40(62.5%)	68 (77.27%)*	43 (30.71%)	

**ACE genotypes**	II	4(12.5%)	1(2.27%)	38(54.29%)	* OR = 0.012, 95% CI (0.001-0.095) *X^2 ^*= 36.97, P = 0.0001
		
	ID	16(50%)	18(40.91%)	21 (30%)	
		
	DD	12(37.5%)	25(56.82%)*	11 (15.71%)	

**AT1R Alleles**	A	40(62.5%)	52(59.09%)	114 (81.42%)	*For C allele MHD Carriers: OR = 0.33 95%CI(0.18-0.60) *X^2 ^*= 13.61, P = 0.0001
		
	C	24(37.5%)	36(40.91%)*	26 (18.58%)	

**AT1R genotypes**	AA	12 (37.5%)	16(36.37%)	48(68.57%)	*OR = 0.23,95%CI (0.10-0.51) *X^2 ^*= 13.63, P = 0.0001
		
	AC	16 (50%)	20 (45.45%)	18 (25.72%)	
		
	CC	4(12.5%)	8 (18.18%)*	4(5.71%)	

Data was evaluated by the gene counting method. Test for allele frequency difference Chi-square tests were used. Values were presented as percentage. CT = conservative treatment, MHD = maintenance hemodialysis, ACE = angiotensin converting enzyme, AT1R = angiotensin II type 1 receptor.

There was a significant difference between the MHD group and the controls as regard to DD genotype (*X^2 ^*= 36.97, P = 0.0001). This may suggest that patients with DD genotype are at high risk of developing renal disease (OR = 0.012, 95% CI = 0.001-0.095). Further, we have analyzed the data by pooling the II genotype with DD genotype. The genotypic level was also visible at the allelic level as D allele was found in a higher frequency in MHD patients than in the controls. (*X^2 ^= 46.89, P = 0.0001*, OR = 0.13, 95% CI = 0.07-0.24). The MHD group showed an increased frequency of the C allele(*X^2 ^*= 13.61, P = 0.0001, OR = 0.33, 95%CI = 0.18-0.60) and the homozygous genotype CC of the AT1RA1166C polymorphism compared to the controls *(X^2 ^*= 13.63, P = 0.0001, OR = 0.23, 95%CI = 0.10-0.51).No significant differences were observed between CT patients and the controls as regards to ACE or AT1RA1166C genotypes or alleles.

### Clinical characteristics of CKD patients with different ACE and AT1R genotypes

In order to assess the cumulative effect of ACE gene polymorphism with other risk factors; we compared various clinical parameters of the CKD patients between two genotypic groups, DD and ID+II. Interestingly, plasma ACE level was strongly associated with the ACE I/D polymorphism, with an additive effect of the D alleles. Serum ACE activity was found to be higher in the DD group than in the II+ DI group (p = 0.02) (Table 3).

**Table 3:** Clinical characteristics of CKD patients with different ACE genotypes

	DD (n = 37)	II+ID (n = 39)	P-value
**Age(Years)**	11.21 ± 3.34	10.91 ± 4.51	0.78

**SBP(mmHg)**	130.96 ± 17.43	120.00 ± 14.04	0.04*

**DBP(mmHg)**	84.00 ± 12.24	84.00 ± 11.21	0.65

**Total cholesterol(mg/dl)**	187.71 ± 57.49	173.67 ± 38.91	0.25

**Triglyceride(mg/dl)**	154.15 ± 74.29	148.44 ± 40.81	0.36

**HDL-cholesterol(mg/dl)**	27.46 ± 12.81	24.13 ± 11.44	0.65

**Creatinine(mg/dl)**	6.20 ± 1.46	6.69 ± 1.41	0.42

**Urea(mg/dl)**	72.09 ± 22.35	68.87 ± 15.65	0.85

**hs-CRP(mg/dl)**	3.57 ± 3.37	2.71 ± 4.00	0.63

**CK-MB(ng/ml)**	5.78 ± 1.61	5.01 ± 1.21	0.63

**Hypertensive%**	89.19%	61.54%	0.02*

**ACE activity(IU/l)**	77.29 ± 58.10	50.10 ± 23.18	0.02*

**Left ventricular mass index (g/m^2.7^)**	55.69 ± 10.47	51.38 ± 9.72	0.34

**Severe left ventricular hypertrophy, n (%)**	16(43.24%)	15(38.46%)	0.36

Significance was estimated using independent t-test. Data was means ± SD .SBP = systolic blood pressure, DBP = diastolic blood pressure, hs-CRP = high sensitivity C - reactive protein, CK-MB = creatine kinase-MB fraction. P < 0.05 was considered significant.

When we compared the number of hypertensive patients between the two sub groups it was noticeably evident that ~89% of the DD genotype patients were hypertensive as compared to the 61% of II+ID genotype group (P = 0.02). The results further confirmed the association of DD genotype with the hypertensive state and implicate a strong possible role in renal damage.

We pooled patients homo- and heterozygous for the C allele for comparison with the AA homozygotes. When serum creatinine and urea levels were compared between the two sub groups, the difference was found to be significant as regards to urea level (P = 0.04). Patients that carry C- allele had the highest ACE activity, while those carrying A-allele had the lowest (P = 0.04) (Table 4).

**Table 4:** Clinical characteristics of CKD patients with different AT1Rgenotypes

	AA (n = 28)	AC+CC (n = 48)	P- value
**Age(Years)**	10.13 ± 4.15	10.97 ± 3.36	0.45

**SBP(mmHg)**	128 ± 17.81	120.5 ± 15.56	0.85

**DBP(mmHg)**	83.33 ± 12.91	81.10 ± 11.56	0.52

**Total cholesterol(mg/dl)**	202.44 ± 55.25	177.50 ± 49.51	0.85

**Triglycerides(mg/dl)**	133.43 ± 71.98	153.08 ± 59.95	0.47

**HDL-Cholesterol (mg/dl)**	26.18 ± 11.54	30.25 ± 18.09	0.36

**Creatinine(mg/dl)**	5.64 ± 1.63	6.72 ± 1.29	0.23

**Urea(mg/dl)**	60.00 ± 12.85	80.65 ± 21.36	0.04*

**hs-CRP, mg/dl**	4.28 ± 4.06	2.70 ± 2.91	0.43

**CK-MB(ng/ml)**	5.14 ± 1.10	5.58 ± 1.29	0.52

**Hypertensive%**	57.14%	47.92%	0.65

**ACE activity(IU/l)**	61.85 ± 54.91	84.26 ± 55.89	0.04*

**Left ventricular mass index(g/m^2.7^)**	53.88 ± 9.33	52.33 ± 11.02	0.52

**Severe left ventricular hypertrophy, n (%)**	11(39.29%)	20(41.76%)	0.62

Significance was estimated using independent t-test. Data was means ± SD. SBP = systolic blood pressure, DBP = diastolic blood pressure, hs-CRP = high sensitivity C- reactive protein, CK-MB = creatine kinase-MB fraction. *P < 0.05 was considered significant.

A high significant inverse correlation was found between serum TG level and the equilibrated KT\V (r = -0.72, P = 0.002). A positive correlation was found between serum CK-MB level and serum urea level (r = 0.50, P = 0.005). DBP was found to be positively correlated with serum hs-CRP level (r = 0.33, P = 0.03).

### Correlation between LVMI and different cardiovascular risk factors

LVMI was positively correlated with indexed SBP (r = 0.42, P = 0.008), indexed DBP (r = 0.58, P = 0.0001) and CK-MB levels (r = 0.36, P = 0.04) (Table 5).

**Table 5:** Correlations between LVMI and different variables

	LVMI	
	
	r	P- value
**Age**	-0.04	0.32

**SBP**	0.42	0.008**

**DBP**	0.58	0.0001**

**Urea**	0.02	0.35

**Creatinine**	0.23	0.42

**hs-CRP**	0.25	0.36

**CK-MB**	0.36	0.04*

**ACE- activity**	0.10	0.21

Correlation was performed by Pearson's analysis. **P < 0.01 and *P < 0.05 was considered significant.

Multiple linear regression analysis demonstrated that the risk factors for hypertension of patients with CKD were serum urea (ß = 0.20, P = 0.04), serum hs-CRP level (ß = 0.32, P = 0.04) and CK-MB level (ß = 0.25, P = 0.02). C-allele was independently associated with hypertension (ß = 0.32, P = 0.04). On correlating LVMI to other variables, serum CK-MB level (ß = 0.30, P = 0.04), serum TG concentration (ß = 0.66, P = 0.04), serum urea level (ß = 0.81, P = 0.02), serum creatinine concentration (ß = 0.51, P = 0.03) and indexed DBP (ß = 0.63, P = 0.0001) were independently associated with LVMI. No significant interaction was observed between D- allele and C-allele in relation to LVMI (ß = 0.01, P = 0.53 and ß = 0.08, P = 0.66 respectively) (Table 6).

**Table 6:** Risk factors affecting hypertension and LVMI in CKD patients based on multiple linear regression analysis

Dependent variables		ß	Unstandardized B	95%CI for ß	P-value
**Indexed SBP**	Serum urea	0.20	7.36	1.55-8.63	0.04*
	Serum creatinine	0.02	1.62	5.76-9.01	0.63
	
	ACE activity	0.01	0.07	0.09-0.23	0.37
	
	D-allele	0.09	0.90	0.96-1.32	0.35
	
	C-allele	0.32	8.35	1.53-8.65	0.04*
	
	hs-CRP	0.32	9.52	1.54-9.61	0.04*
	
	CK-MB	0.25	7.35	1.65-7.68	0.02*

**LVMI**	D-allele	0.01	1.23	12.40-15.36	0.53
	
	C-allele	0.08	2.45	13.74-18.66	0.66
	
	hs-CRP	0.09	1.27	7.17-9.71	0.58
	
	CK-MB	0.30	9.63	1.64-7.61	0.04*
	
	TG	0.66	6.50	0.98-2.50	0.04*
	
	Urea	0.81	5.42	1.76-8.17	0.02*
	
	Creatinine	0.51	5.41	0.99-9.83	0.03*
	
	Indexed DBP	0.63	5.63	0.46-1.44	0.0001**

ACE = angiotensin converting enzyme, hs-CRP = high sensitivity c-reactive protein, CK-MB = creatine kinase-MB fraction, TG = triglycerides, DBP = diastolic blood pressure, CI = Confidence Interval. **P < 0.01 or *P < 0.05 was considered significant.

## Discussion

Renal disease progression resulted from the interaction of multiple environmental and genetic factors. Several studies had shown a relationship between genetic variants of the renin-angiotensin system genes and renal diseases as well as the rate of progression of renal damage (reviewed in [20]).

The current data demonstrated an association between the ACE, and AT1R gene polymorphisms and advanced CKD in children undergoing MHD compared with conservative treatment. The I/D polymorphism of the ACE gene and plasma concentration were studied as a cluster of cardiovascular risk factors that could contribute to excess metabolic cardiovascular and renal risks in MHD patients compared with patients undergoing CT. Several reports linked this polymorphism to the development and progression of chronic renal diseases of different etiologies [30-33].

Our study revealed highly significant differences in the presence of DD genotype and D allele of ACE gene in MHD patients than in normal controls. These differences might validate that the ACE gene polymorphism is an important genetic determinant of non-diabetic nephropathies. D allele of ACE gene might confer a high risk of developing renal diseases and this association was highly compounded when D allele was present in homozygous state. Even inclusion of the heterozygous ID state known to have intermediate levels of ACE production along with the DD genotype depicted a high risk of renal failures. Therefore, the finding that ACE DD genotype and D allele was associated with renal ESRD is likely to be true for pediatric populations [34]. There was no significant difference between CT patients and the controls as regards to ACE DD genotype or D allele. This may be due to small sample size of CT group.

Our results were free of genotyping errors/mistakes in data manipulation ("blind" genotyping or validation using different methodologies) and were in accordance with results of others as Settin et al. [35] with his study on 79 Egyptian myocardial infarction cases, he found that cases had a higher frequency of DD (29.1%) and ID (62.0%) genotypes than II (8.9%) genotype, with a higher frequency of D allele than I allele (64.4% vs. 33.6%). Compared to controls, cases had a significantly higher frequency of ID genotype (62.0% vs. 47.5%, P < 0.05) and he concluded that the angiotensin-converting enzyme gene I/D polymorphism is probably a risk factor for ischemic heart disease among Egyptian cases. Also in a study done by Ketat et al. [36] he found that in Egyptian patients with diabetic nephropathy, ID and DD genotypes were present in 20% and 25% respectively as compared to 2% and 0% in controls respectively. Thus, D allele was present in 45% of the Egyptian patients as compared to 2% of normal controls. He concluded that there is a positive association between the D-allele and the development of diabetic nephropathy in Egyptians. There are many other Egyptian studies as Fahmy et al. [37] who reported that idiopathic nephrotic syndrome is associated with a higher incidence of DD genotype, especially in non-steroid sensitive patients and DD genotype may play a role in the clinical response to steroid. Also Morsy et al. [38] who concluded that patients with rheumatic heart disease (RHD) had a higher ACE-DD genotype than normal control. ACE-DD genotype might be a risk factor for RHD in Egyptian children.

We postulated that DD genotype confered a greater role in hypertensive state as ~89% of DD genotype patients were hypertensive and this phenomenon might have been the major factor behind the association of ACE genotypes and ESRD pediatric patients.

Hypertension being a complex polygenic disorder is often regarded as a physiological state affected by, "Genetic Predisposition" which highlights the presence of heritable allelic differences in the genes coding/associated with different components of RAS. Such differences result into differential transcript and protein expression accounting for different rates of progression of hypertension and other related diseases mainly, renal failures [35].

The DD genotype had unanimously been shown to have increased serum ACE production and activity while II and ID genotypes produced low and intermediate levels of proteins respectively [35]. In this study, we observed that plasma ACE level was strongly associated with the ACE D/D polymorphism and the effect of the D allele on plasma ACE activity was additive. Various reports are available supporting that how the presence of DD genotype operates at cellular level leading to hypertensive state and renal diseases [35-38].

Association between hypertension and ACE gene polymorphism had not been found in the general population, in some particular conditions, such as malignant hypertension, the D allele had been shown to be a significant risk factor [39]. In dialysis patients, blood pressure can be controlled by sodium and fluid removal. Carriers of the D allele seemed to be less sensitive to sodium state than I carriers and could therefore be less responsive to sodium removal by ultra-filtration in dialysis [18]. Several renin angiotensin system polymorphisms alter the homeostasis to an abnormal state. Similarly, other genes such as nephrin (NPHS1) and podocin (NPHS2) contribute to the loss of renal function during renal diseases. In a study done by Anbazhagan et al. [4] ACE-DD genotype showed a higher level of systolic pressure with a median of 166 mmHg (P < 0.05) when compared to II and ID genotypes and two heterozygous conditions of NPHS2-R229Q polymorphism were found among 105 CKD patients.

The interesting finding of our study was the association of the AT1RA1166C genotype with the development of renal disease and progression to end-stage renal failure. This confirmed a previous result [40]. We observed a significant difference in the frequency of the C allele and CC homo-zygotes in MHD patients than in controls. Due to a small number of patients with the CC genotype, AC and CC genotypes were pooled for the renal deterioration analysis. Patients carrying the C allele showed more a rapid deterioration of renal function (urea concentration) than those with the AA genotype. The mechanism by which the AT1RA1166C polymorphism affects the development of renal disease and its progression to ESRD remains to be elucidated. It is possible that predisposition to renal disease is related to genetic variability in the sensitivity of target tissues to angiotensin II whose actions are mediated by the AT1R receptor. The studied polymorphism is located in the 3' untranslated region of the gene and is apparently a nonfunctional mutation [41]. It may be linked, however, to an unidentified functional mutation in the AT1R gene or in another closely linked gene possibly located in regulatory regions and involved in the development and progression of renal damage.

The present study revealed that patients carrying C- allele had the highest ACE activity, while those carrying A-allele had the lowest. Inhibition of the RAS, either through reducing the production of angiotensin II with ACEI or by blocking the action of angiotensin II at the AT1R receptor level with A II-type 1 receptor blockers (ARBs), is particularly effective at preventing renal injury [41].

On correlating indexed SBP to different cardiovascular risk markers by multiple linear regression analysis, we found that C-allele, serum urea, hs-CRP and CK-MB were variables that were independently associated with indexed SBP. In hypertensive patients it is suggested that the combination of DD polymorphism type and AC/CC for AT1R gene, could contribute in a synergistic way to organ damage. The AT1R mediates the more deleterious effects of angiotensin II--that is, cardiac and vessel hypertrophy including extracellular matrix production. In addition to the conversion of angiotensin I to angiotensin II, ACE inactivates the vasodilator peptide bradykinin [20]. Studies on the general population and in selected families have shown that the AT1R gene polymorphism may increase the susceptibilities to essential hypertension [31]. TheAT1R A1166C polymorphism has been found to be associated with higher angiotensin II sensitivity in hypertensive patients on a high-salt diet [42].

The relationships between the ACE gene polymorphism and LV mass and remodeling were extensively investigated in different populations [42,43]. Theoretically DD genotype, which is associated with increased ACE activity, together with CC genotype may further promote cardiac growth and remodeling and contribute to the higher prevalence of LVH among patients with DDCC genotypes [42]. Di Mauro et al. evaluated the role of angiotensin type 1 receptor gene (AGTR1) and ACE polymorphisms in LVH in endurance athletes. The group DD showed a slightly higher prevalence of LVH than group ID. The highest LVMI was found in 15 athletes with ACE-DD and AGTR1-AC/CC genotypes and the lowest value of LVMI was found in the case of ACE-ID and AGTR1-AA. The presence of ACE-DD + AGTR 1 + AC/CC was strongly associated with LVH [43]. Also, Hernandez et al. reported that ACE/DD genotype was associated with the extent of exercise-induced left ventricular growth in endurance athletes regardless of other known biologic factors [44]. Takami et al. suggested that gene polymorphisms of both angiotensin II receptors are not directly involved in the increase of genetic risk for hypertension, but the AT1R might contribut to the increase of LVM [45].

In the present study LVMI was not associated with any of the polymorphisms examined. The absence of a gene dosage effect on LVMI may be because (1) tissue ACE activity may be more important and may be influenced by gene polymorphism differently from serum ACE activity and (2) there may be no mechanistic relationship between the ACE polymorphism and LVMI.

Some reports indicated a high prevalence of LVH in children on dialysis, as identified in adults. However, the mean LVMI was higher in our patients than in the patients in other pediatric studies [46,47]. Two most important reasons for this could be that mean CK-MB level and mean BP were higher in our patients due to non compliance of patients to anti-hypertensive treatment and salt/fluid restriction [46,47]. Control of hypertension might be an important factor in regression of LVH in ESRD. In the present study, linear regression analysis revealed that indexed DPB, TG concentration, serum urea, creatinine and CK-MB levels were the most important independent contributors to the risk of ESRD-related LVH. Martin et al. [48] stated that LVH which contributes to myocardial ischemia is found to be a highly predictive of high serum levels of cardiac markers as CK-MB. hs-CRP is frequently considered as an epiphenomenon rather than a pathogenic mechanism in development of LVH [49]. Finally, according to our data hs-CRP is a risk marker of CVD in children with ESRD. Our result was similar to a previous study [49].

There were some limitations in this study. The small sample size of the patients and this leads to low statistical power and insignificant difference between CT patients and the controls as regards to ACE and AT1RA1166C gene polymorphisms. Also, only one centre is included in the study. Further large study on the pediatric Egyptian population from different renal centres will be done for better interpretation for the role of ACE gene polymorphism on the progression of renal failure.

## Conclusion

ACE gene polymorphism appeared to be an important genetic determinant in causation and progression of renal diseases and DD genotype was found to be significantly associated with advanced ESRD in children. Our results suggested that the CC/AC genotype might serve as a predictor of an early pediatric ESRD and could in the future become an important part of the clinical process of renal risk identification. Further studies in this regard will open a plethora of options like timing, type and doses of anti-hypertensive therapy. Incorporation of such approaches will allow an advance anticipation of the clinical outcome and can lead to a shift from "One treatment fits all" approach.

## Competing interests

The authors declare that they have no competing interests.

## Authors' contributions

MFE, SMS and HMB carried out all samples collection and patients work up. MFE has interpretated the data, performed the statistical analysis and has written the manuscript. HMK was involved in the patients work up. EAE, NAK, EHT and DAH have performed the immunoassay and the gene polymorphism determination. All authors read and approved the final manuscript.
